# Development of SiC Nanoparticles and Second Phases Synergistically Reinforced Mg-Based Composites Processed by Multi-Pass Forging with Varying Temperatures

**DOI:** 10.3390/ma11010126

**Published:** 2018-01-13

**Authors:** Kaibo Nie, Yachao Guo, Kunkun Deng, Xiaojun Wang, Kun Wu

**Affiliations:** 1 College of Materials Science and Engineering, Taiyuan University of Technology, Taiyuan 030024, China; ycguotyut@126.com (Y.G.); jamsdk@163.com (K.D.); 2 Shanxi key laboratory of advanced magnesium-based materials, Taiyuan University of Technology, Taiyuan 030024, China; 3 School of Materials Science and Engineering, Harbin Institute of Technology, Harbin 150001, China; xjwang@hit.edu.cn (X.W.); wukunhit@163.com (K.W.)

**Keywords:** magnesium matrix composite, SiC nanoparticles, second phases, multi-pass forging, mechanical properties

## Abstract

In this study, SiC nanoparticles were added into matrix alloy through a combination of semisolid stirring and ultrasonic vibration while dynamic precipitation of second phases was obtained through multi-pass forging with varying temperatures. During single-pass forging of the present composite, as the deformation temperature increased, the extent of recrystallization increased, and grains were refined due to the inhibition effect of the increasing amount of dispersed SiC nanoparticles. A small amount of twins within the SiC nanoparticle dense zone could be found while the precipitated phases of Mg_17_Al_12_ in long strips and deformation bands with high density dislocations were formed in the particle sparse zone after single-pass forging at 350 °C. This indicated that the particle sparse zone was mainly deformed by dislocation slip while the nanoparticle dense zone may have been deformed by twinning. The yield strength and ultimate tensile strength of the composites were gradually enhanced through increasing the single-pass forging temperature from 300 °C to 400 °C, which demonstrated that initial high forging temperature contributed to the improvement of the mechanical properties. During multi-pass forging with varying temperatures, the grain size of the composite was gradually decreased while the grain size distribution tended to be uniform with reducing the deformation temperature and extending the forging passes. In addition, the amount of precipitated second phases was significantly increased compared with that after multi-pass forging under a constant temperature. The improvement in the yield strength of the developed composite was related to grain refinement strengthening and Orowan strengthening resulting from synergistical effect of the externally applied SiC nanoparticles and internally precipitated second phases.

## 1. Introduction

Light-weight magnesium alloy with high strength is potential choice for the applications in the fuel-intensive automotive and aerospace sectors, as well as for electronic components and biomedical implants, due to its low mass density, high stiffness and environmental friendliness [1,2,3]. The past decades have seen more and more interest in preparing magnesium alloy with excellent mechanical properties that are attractive for the expected functionalities. Some strategies including grain refining, alloying, composites and thermal-mechanical treatment have been used to design Mg-alloy microstructures and improve strength [4,5,6,7]. In fact, combinations of the above strategies may be beneficial to achieve desired properties. Previous research has shown that designed magnesium matrix composites with refined grains can enhance both strength and ductility [8,9,10]. Among the various reinforcements, discontinuous ceramic nanoparticles have outstanding advantages such as chemical inertness, isotropic properties and excellent mechanical properties, which make them attractive for the preparation of magnesium matrix composites [11,12,13,14]. To obtain defect-free microstructure, as well as better mechanical properties, reinforcement particles should be homogeneously distributed in the matrix. In this situation, cost-effective fabrication technology should be developed to incorporate and disperse nano-sized ceramic particles homogeneously in magnesium alloy. In our previous study, the combination of semisolid stirring and ultrasonic infiltration was successfully adopted to prepare the SiC nanoparticles reinforced magnesium matrix composites [15].

With regard to extensive conducted studies related to the fabrication of nanoparticles reinforced magnesium matrix composite, only a few researches focus on the secondary thermo mechanical processing of as-cast composites [16,17,18]. Liu et al. reported that after hot rolling there was an obvious increase in the yield strength for the AZ31 based composite reinforced with SiC nanoparticles, as compared with as-cast counterparts [16]. Compared with the matrix alloy, the ultimate tensile strength, yield strength and ductility of the nano-SiCp/Mg composites enhanced after hot extrusion as revealed by Choi et al’s experiment [17]. Our previous study indicated that hot extrusion of the nano-SiCp/AZ91 composite could lead to the occurrence of extensive dynamic recrystallization and the significant refinement of matrix microstructure [18]. Therefore, the application of a thermo mechanical process can further improve the quality of the nanoparticles reinforced magnesium matrix composite. With respect to bulk materials, it is more effective to obtain exceptional grain refinement using severe plastic deformation (SPD) with large accumulated strain and abundant material flow, which is different from that by conventional thermal deformation [19,20,21,22]. Among various SPD procedures, multi-pass forging is particularly promising for fabricating large piece of material, an advantage that is mainly attributed to its special process [23,24,25,26,27]. The sample shape can be kept during multi-pass forging, while thickness and diameter usually reduce after conventional thermal deformation. Besides, multi-pass forging is also suitable for brittle materials because deformation temperature is relatively high and applied pressure is small. Moreover, compared with the conventional processing method anisotropy in the sample could be alleviated after multi-pass forging due to the loading applied in three directions [24]. Currently, submicrometer or nanometer grains have been successfully acquired in pure metals and metallic alloys through the application of multi-pass forging [27]. However, very limited reports are available on the influence of multi-pass forging on the microstructure evolution and property change of the magnesium matrix composites [28,29,30]. Most attention has been paid to the multi-pass forging of conventional micro-particles reinforced magnesium matrix composites [29,30]. In addition, as the sizes of reinforcements are less than 1 μm, dislocation glide by Orowan mechanism can be hindered by nano-sized particles, leading to significant improvement in strength [31]. The anticipated effect of nanoparticles is thought to be different from micro-particles regarding the microstructure evolution and property change of matrix alloy in the magnesium matrix composites during multi-pass forging. Moreover, research indicated that second phase strengthening is a main way to enhance the mechanical properties of magnesium alloy, while the precipitation of second phases is sensitive to the change of deformation temperature [32]. However, literature searches show that there is a lack of systematic study on the SiC nanoparticles and second phases synergistically enhanced magnesium matrix composite especially when fabricated by multi-pass forging with varying temperatures.

Therefore, the synergistical influence of nanoparticle addition and second phases on tensile strength at room temperature and the microstructure of matrix alloy during multi-pass forging with varying temperatures is investigated in the paper. The nanoparticle reinforced composite were initially prepared with semisolid stirring and ultrasonic infiltration followed by solution treatment and multi-pass forging with varying temperatures. The relationship between microstructures and deformation behaviors of the composites is analyzed, which may open a new approach for further studies on magnesium matrix composite.

## 2. Experimental Procedures

### 2.1. Fabrication of Nano-SiCp/AZ91 Composite

The matrix of the composite is AZ91 alloy with nominal composition of Mg-9.07Al-0.68Zn-0.21Mn, which is provided by Northeast Light Alloy Company Limited, Harbin, China. The reinforcement is nano-sized SiC particles with an average dimension of 60 nm, which is supplied by Hefei Kaier Nanometer Energy & Technology Company Limited, Hefei, China. SiC particles with volume fraction of 1% were added into matrix alloy using semisolid stirring and ultrasonic infiltration. A detailed description of the used fabrication process is given by Nie et al. [15]. First, semi-solid AZ91 alloy melt was kept at a temperature of 590 °C with a shielding gas of CO_2_/SF_6_ and mechanical stirring was adopted to add SiC nanoparticles. Secondly, the melt was heated up to 700 °C and processed by applying the ultrasonic vibration. Finally, liquid composite was poured into a preheated mold with a temperature of 450 °C and solidified under pressure. It must be pointed out that there was no ultrasound applied during solidification. In order to minimize the influence of the Mg_17_Al_12_ phase, as-cast ingots were solution treated at 415 °C for 24 h before multi-pass forging.

### 2.2. Multi-Pass Forging of Nano-SiCp/AZ91 Composite

The as-cast composite ingot was cut into rectangular billet specimens with a size of 30 × 30 × 60 mm through the electrical discharge machining method. The multi-pass forging was performed on a press with a load limit of 2000 kN. The deformation temperatures changed from 250 °C to 400 °C while punch velocity was 15 mm s^−1^. The original shape of the billets was maintained constant on the whole at the end of the multi-pass forging although the direction of the load was rotated 90° between passes. To create a uniform temperature distribution, all of the specimens were heated to the desired deformation temperatures performed in a resistance furnace. The strain for each pass forging was 0.693 [32]. A graphite-based mixture was utilized as a lubricant at different deformation temperatures.

### 2.3. Microstructures Characterization

By means of optical microscopy (OM; Shanghai Optical Instrument Factory, Shanghai, China), scanning electron microscopy (SEM; Tescan, Brno, Czech Republic), and transmission electron microscopy (TEM; JEOL Ltd., Tokyo, Japan), microstructures of the composites were examined. Samples for metallographic observation and SEM were prepared by the polishing machine and then etched in acetic picral (5 mL acetic acid + 6g picric acid + 10 mL H_2_O + 100 mL ethanol (95%)). Image-Pro Plus image analysis software (Ipwin32, Media Cybemetics Co., Rockville, MD, USA) was used to analyze the grain size, and the phase identification of the composites was carried out on an energy dispersive spectroscope (EDS). The thickness of the TEM specimens was reduced to less than 50 μm by manual grinding. Then a disk with diameter of 3 mm was sliced and ion beam thinned. 

### 2.4. Tensile Test

In an uniaxial tension experiment, the mechanical properties of the composite reinforced with SiC nanoparticles were determined using an Instron-1186 tension machine. Flat dog-bone samples with gage size of 15 × 6 × 2 mm perpendicular to the last compression axis were machined from billet specimen by numerically controlled wire cutting machine. The initial strain rate for tensile test was 8.33 × 10^−4^ (s^−1^). In the present work, the values of tensile properties were calculated according to three repeated tensile tests.

## 3. Results and Discussion

### 3.1. Microstructure and Tensile Properties after Single-Pass Forging at Different Temperatures

**Figure 1:**
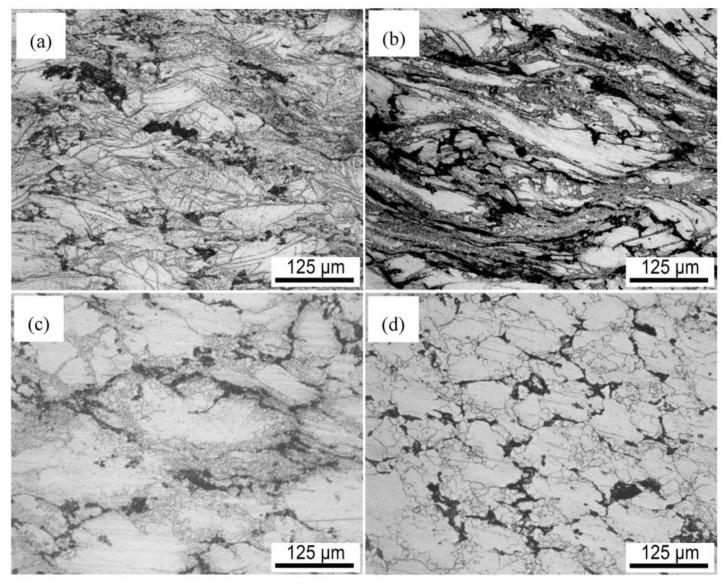
Optical microscopy (OM) images of nano-SiCp/AZ91 composite after single-pass forging at different temperatures: (**a**) 250 °C; (**b**) 300 °C; (**c**) 350 °C; (**d**) 400 °C.

**Figure 2:**
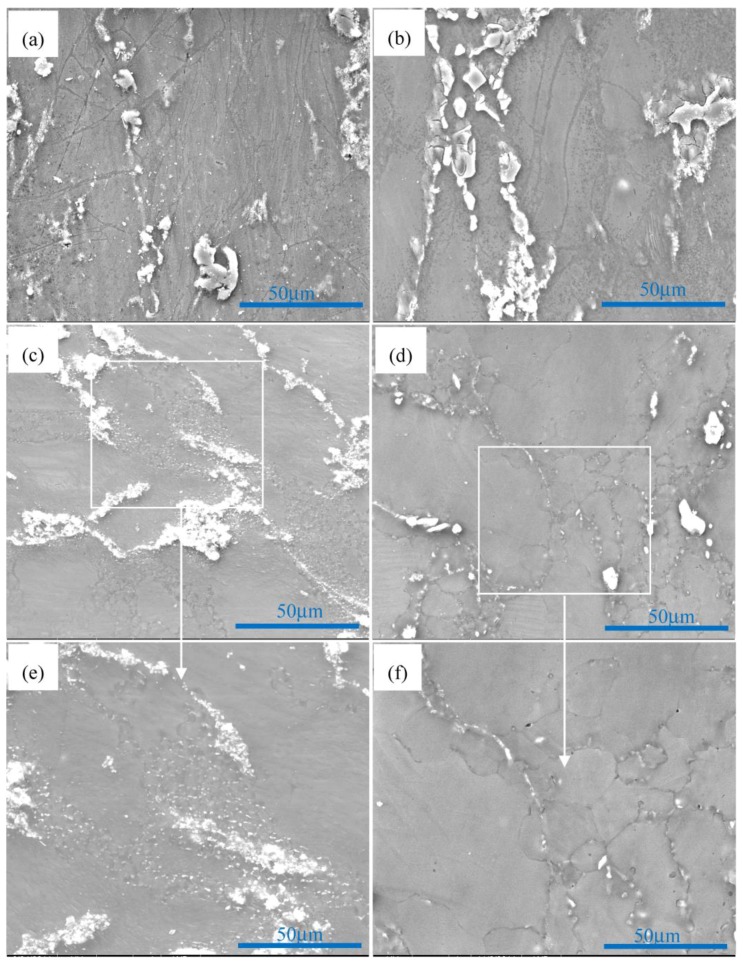
Scanning electron microscopy (SEM) of the nano-SiCp/AZ91 composites after single-pass forging under different temperatures: (**a**) 250 °C; (**b**) 300 °C; (**c**,**e**) 350 °C; (**d**,**f**) 400 °C.

The optical microstructure of the nano-SiCp/AZ91 composite subjected to single-pass forging at different temperatures is given in Figure 1. In our previous studies, after solution treatment the microstructure for the as-cast composite was composed of a α-Mg matrix with some SiC nanoparticles distributed along matrix grain boundaries [15]. After single-pass forging at 250 °C, as shown in Figure 1a, a high amount of twins can be observed. After single-pass forging at 300 °C, as shown in Figure 1b, the trend of dynamic recrystallization (DRX) is very low while distorted original grains are found. With the deformation temperature increasing up to 350 °C, as shown in Figure 1c, fine grains appear along the initial grains occupying a significantly larger area fraction, and showing a typical DRX characteristic. When the deformation temperature was increased to 400 °C, the grain size of the matrix alloy is further reduced, as shown in Figure 1d. To clarify the microstructure more clearly, the results of SEM observation for the composites after single-pass forging with the deformation temperatures ranging from 250 °C to 400 °C are presented in Figure 2. According to Figure 2a, there are no DRX grains detected in the composites after single-pass forging at 250 °C. When the deformation temperature is 250 °C, the magnesium matrix deforms essentially by twinning under a low deformation temperature, resulting in the formation of twins (Figure 1a and Figure 2a). Research indicated that when the AZ91 alloy was subjected to SPD at 300 °C, twinning can be changed to DRX and DRX could occur in this situation [33]. Thus, after single-pass forging at 300 °C only a small amount of DRX grains are formed by a detailed view of the composite as shown in Figure 2b. Under higher deformation temperatures of 350 °C or 400 °C, as shown in Figure 2c,d, some SiC nanoparticle dense zones and fine grains along initial coarse grain boundaries can be found in the composites. In general, there is high-density dislocation tangle along the vicinity of the initial grain boundaries, which are preferred sites for DRX grains formation [34]. Therefore, after single-pass forging at 350 °C, the fine grains nucleate along initial grain boundaries (Figure 1c and Figure 2e), which indicates that DRX occurred. The micron-SiC particles reinforced magnesium matrix composites after single-pass forging were recrystallized completely [29,30] and were also different from the present nano-sized particles reinforced magnesium matrix composites. This is because grain refinement due to particle-stimulated DRX during hot deformation is unlikely when the particle size is less than 1 μm. However, the pinning effect of the nanoparticles with even distribution in the composite on grains of the matrix alloy could generate during the hot deformation process. With increasing the single-pass forging temperature, nanoparticle distribution in the composite has been improved and the growth of DRX grains could be inhibited by increased dispersed nanoparticles (Figure 2e,f). Therefore, the degree of DRX increases for the present composite and the grain size of the matrix decreases with the increase of the single-pass forging temperature.

**Figure 3:**
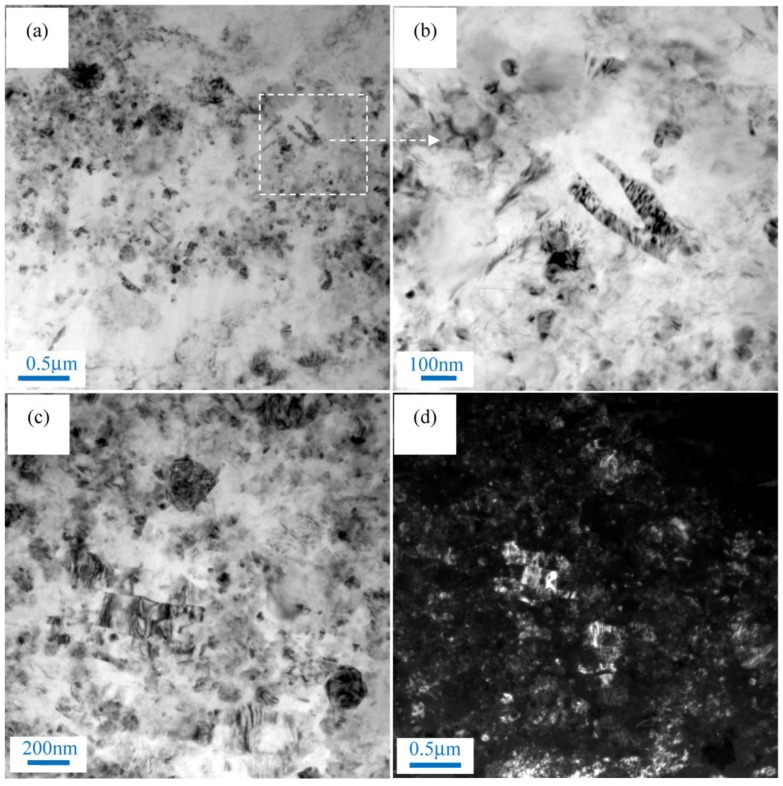
Nanoparticle distribution of nano-SiCp/AZ91 composite after single-pass forging at 350 °C: (**a**) SiC nanoparticles and twin; (**b**) SiC nanoparticles and twin at higher magnification; (**c**) bright field image and (**d**) dark field image of SiC nanoparticles and dynamic recrystallization (DRX) grains.

**Figure 4:**
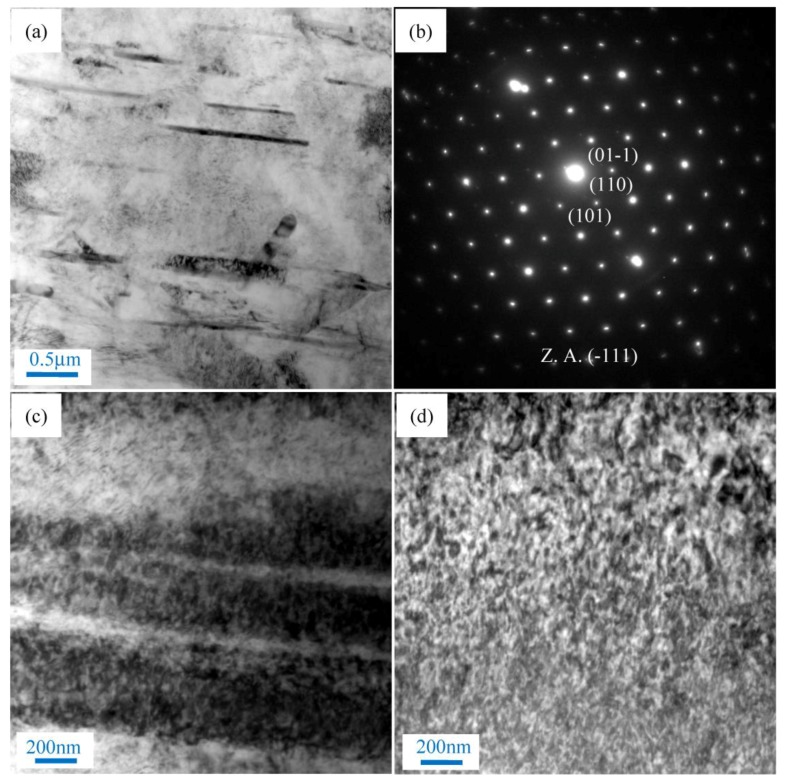
Transmission electron microscopy (TEM) micrographs of nano-SiCp/AZ91 composite after single-pass forging at a temperature of 350 °C: (**a**) precipitated phase; (**b**) electron diffraction patterns of the precipitated phase; (**c**) deformation band; (**d**) dislocation within the deformation band.

Figure 3 shows the nanoparticle distribution of nano-SiCp/AZ91 composite after single-pass forging at a temperature of 350 °C. It can be seen from Figure 3a that the distribution of SiC nanoparticles is relatively homogenous and the dislocation density is low in the SiC nanoparticle dense zone. At higher magnification, a small amount of twins can be found within SiC nanoparticle dense zone. Besides, some DRX appear in the SiC nanoparticle zone as shown in Figure 3c. Dark field image of the composite after single-pass forging at 350 °C is given in Figure 3d. The white areas represent SiC nanoparticles and black areas represent magnesium alloy matrix, which further confirmed that DRX grains are surrounded by SiC nanoparticles. This indicates that the matrix within the SiC nanoparticle dense zone may deform by twin. The TEM micrographs of particle sparse zone in the matrix alloy of nano-SiCp/AZ91 composite after single-pass forging at 350 °C are presented in Figure 4. As shown in Figure 4a, precipitated phases in long strips exist in the composite. The composition for the precipitated phase is confirmed as Mg_17_Al_12_ by electron diffraction patterns (Figure 4b). In addition, it is observed that deformation bands are formed in the matrix alloy as shown in Figure 4c. High density dislocations were found inside the deformation zone (Figure 4d) from the higher magnification of TEM observation. This indicates that the particle sparse zone in the matrix alloy is mainly deformed by dislocation slip under single-pass forging at 350 °C.

**Figure 5:**
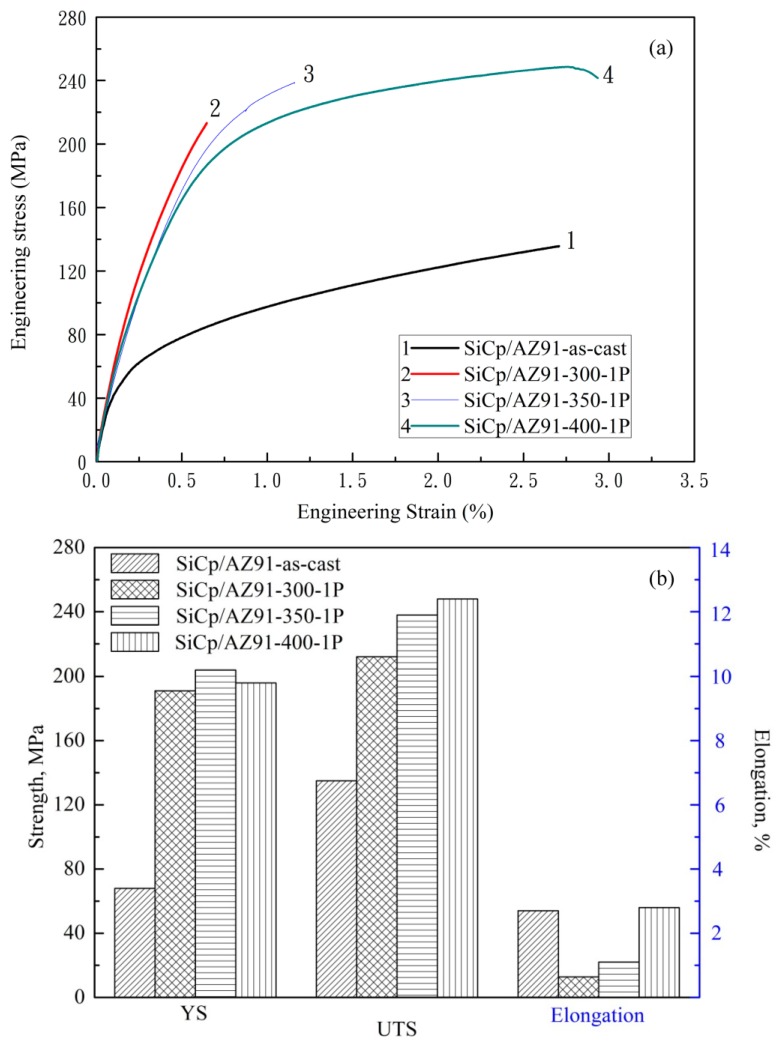
(**a**) The engineering stress–engineering strain curves and (**b**) tensile strength of the nano-SiCp/AZ91 composites after single-pass forging under different temperatures.

Figure 5 shows the engineering stress-engineering strain curves and corresponding strength of nano-SiCp/AZ91 composite after single-pass forging at different temperatures. After single-pass forging at 250 °C premature cracking occurs in the composite, leading to the failure of the composite. Therefore, tensile property of the composite is not provided after single-pass forging at 250 °C. From Figure 5a, the tensile strength of the composites after single-pass forging is obviously increased compared with that of the as-cast composite. In the current work, the effect of the phase Mg_17_Al_12_ on the tensile strength is reduced as the as-cast composite is subjected to T4 treatment before forging. When the deformation temperature is lower than 350 °C, the elongation of the composite after single-pass forging is lower than the as-cast composite. This is due to the existence of SiC nanoparticle dense zones and uncompleted DRX, which have negative effect on the elongation to fracture. With increasing the single-pass forging temperature to 400 °C, the amount of recrystallization grains increases and grain size is gradually decreased (Figure 1). Thus, yield strength and ultimate tensile strength are gradually enhanced for the composites. Besides, with increasing the single-pass forging temperature, improvement of nanoparticle distribution appears in the composite and the dispersed SiC nanoparticles are favorable for improving the elongation to fracture. As a result, the elongation to fracture of the composite is kept compare with the as-cast composite. This indicates that higher forging temperature during the single-pass forging process can not only avoid premature cracking but also achieve grain refinement, which is beneficial for improving the mechanical properties of the present composite.

### 3.2. SiC Nanoparticles and Second Phases Synergistically Reinforced Composite Processed by Multi-Pass Forging with Varying Temperatures

**Figure 6:**
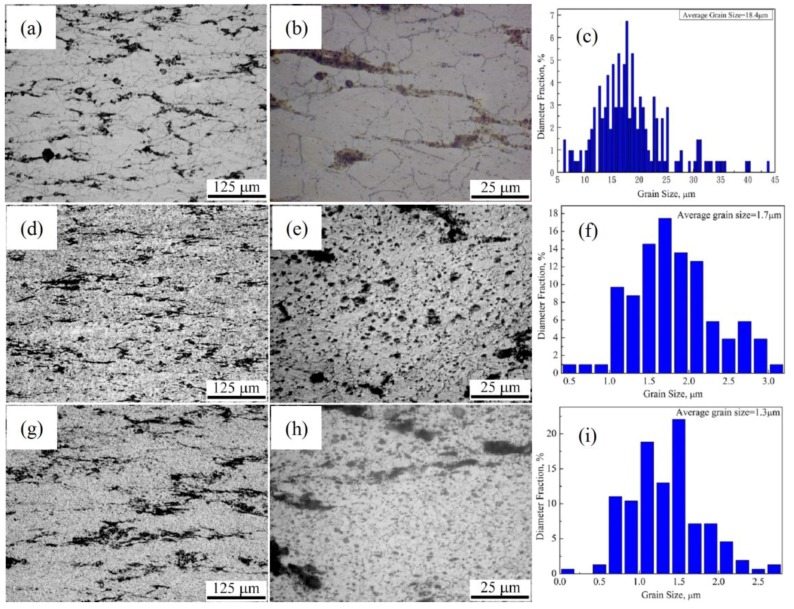
OM images and grain size distribution of nano-SiCp/AZ91 composite after multi-pass forging with varying temperatures: (**a**–**c**) 6 passes at 400 °C; (**d**–**f**) 6 passes at 400 °C and 3 passes at 350 °C; (**g**–**i**) 6 passes at 400 °C, 3 passes at 350 °C and 3 passes at 300 °C.

Based on the above result of the nano-SiCp/AZ91 composite after the single-pass forging process, an initial high forging temperature is favorable for improving the mechanical properties, as it will ultimately enhance the subsequent multi-pass forging deformation capacity. Besides, when the composite was subjected to multi-pass forging with more than three passes under a constant temperature, the grain refinement of the matrix alloy was not obvious with the increase of forging passes as reported in our previous work [35]. In this case, lowering the forging temperature of the subsequent multi-pass forging could be in favor of further grain refinement. Figure 6 shows optical micrographs of and grain size distribution of nano-SiCp/AZ91 composites after multi-pass forging with varying temperatures. As shown in Figure 6a, the DRX behavior of matrix alloy after 6 passes at a constant temperature of 400 °C is more fully compared with that after single-pass forging (Figure 1d). After 6 passes at 400 °C and 3 passes at 350 °C (Figure 6d), grains of matrix alloy is significantly finer than that after 6 passes at 400 °C. The average grain size is 1.7 μm after 6 passes at 400 °C and 3 passes at 350 °C (Figure 6f), and slightly reduced to 1.3 μm after 6 passes at 400 °C, 3 passes at 350 °C and 3 passes at 300 °C (Figure 6g,i). Besides, the distribution of grain size is narrower for 6 passes at 400 °C and 3 passes at 350 °C compared with that for 6 passes at 400 °C, 3 passes at 350 °C and 3 passes at 300 °C. At high magnification, as shown in Figure 6b,e,h, the amount of the precipitated second phases under multi-pass forging with varying temperatures is significantly increased compared with that under multi-pass forging with constant temperature. On the one hand, the increase of multi-pass accumulative deformation can provide the driving force of recrystallization, leading to sustained grain refinement. On the other hand, pinning effect of the SiC nanoparticles as well as the precipitated second phases resulting from decreasing forging temperature could hinder the growth of recrystallization grain. When the forging number is the same, the matrix grain size after 6 passes at 400 °C, 3 passes at 350 °C is decreased compared with that after 3 passes at 400 °C, 3 passes at 350 °C and 3 passes at 300 °C as reported in our previous study [36]. This can be attributed to that initial high deformation temperature is beneficial to break up the nanoparticle agglomeration resulting in the amount increase of nanoparticles, which can abstract the grain growth. When the temperature range of the multi-pass forging is the same, with the increase of the forging passes the grains in the composite are further refined and the microstructure uniformity is improved after 6 passes at 400 °C, 3 passes at 350 °C and 3 passes at 300 °C. 

**Figure 7:**
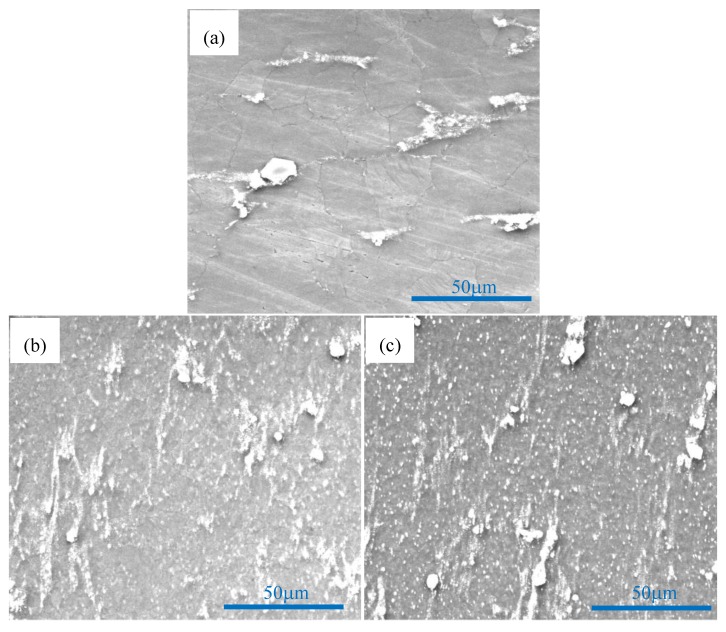
SEM images of nano-SiCp/AZ91 composite after multi-pass forging with varying temperatures: (**a**) 6 passes at 400 °C; (**b**) 6 passes at 400 °C and 3 passes at 350 °C; (**c**) 6 passes at 400 °C; 3 passes at 350 °C and 3 passes at 300 °C.

**Figure 8:**
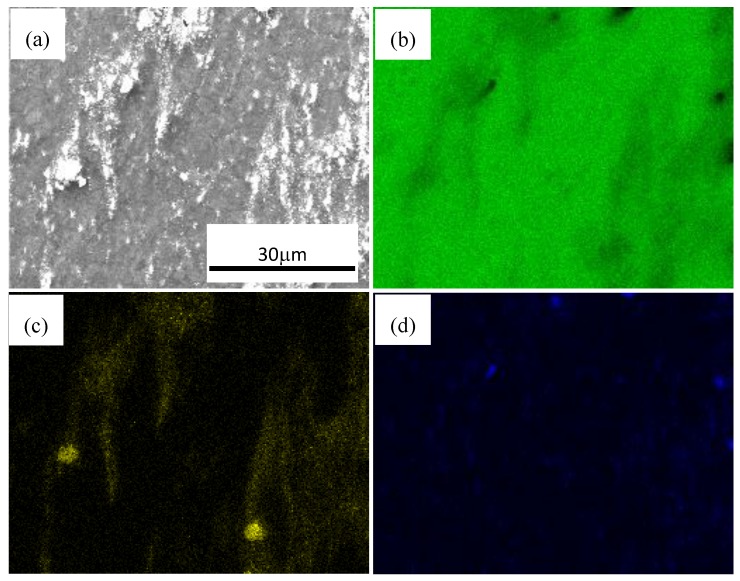
SEM micrographs at high magnification of nano-SiCp/AZ91 composite after 6 passes at 400 °C and 3 passes at 350 °C: (**a**) ditribution of SiC nanoparticles, energy dispersive spectroscope (EDS) of (**b**) Mg K; (**c**) Si K; (**d**) Al K.

**Figure 9:**
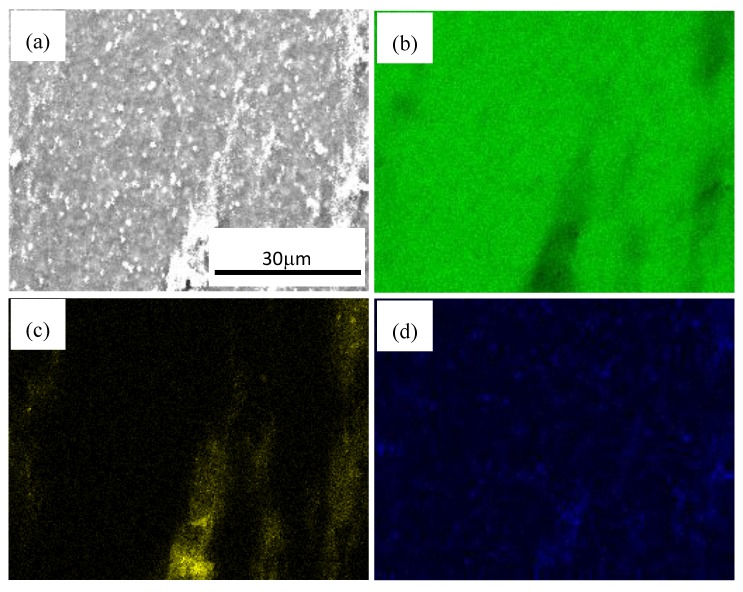
SEM micrographs at high magnification of nano-SiCp/AZ91 composite 6 passes at 400 °C, 3 passes at 350 °C and 3 passes at 300 °C: (**a**) ditribution of SiC nanoparticles, EDS of (**b**) Mg K; (**c**) Si K; (**d**) Al K.

Figure 7 shows SEM photographs of nano-SiCp/AZ91 composite after multi-pass forging with varying temperatures. It can be seen that with increasing the forging number and lowering the deformation temperature, the precipitated second phases Mg_17_Al_12_ in the composite is more and more obvious. For the as-cast AZ91 matrix composite precipitates with a network-like distribution along grain boundaries adversely affect mechanical properties [34]. Before multi-pass forging with varying temperatures, the as-cast composite was solution treated to minimize the influence of Mg_17_Al_12_ phase. Accordingly, it can be deduce that dynamic precipitation of Mg_17_Al_12_ phase is induced on cooling from the deformation temperature. The longer heating time and cooling time accumulates during multi-pass forging with varying temperatures, the more homogenous the solute Al atoms distributes in the magnesium matrix. As a result, more and more Mg_17_Al_12_ phases precipitate in the composite. To observe the distribution of SiC nanoparticles and the phase compositions of black areas, the photos of area scan by SEM and -EDS are obtained as shown in Figure 8 and Figure 9. By analyzing two areas of SEM micrographs in Figure 8a and Figure 9a, the EDS of Mg K, Si K and Al K prove that the components of the particles are SiC nanoparticles as shown in Figure 8b–d and Figure 9b–d. The Si K is uniformly distributed outside a few dense zones in Figure 8 and Figure 9, which can further confirm that the distribution of SiC nanoparticle is even, although some SiC nanoparticles are still in dense zones after multi-pass forging with varying temperatures. Moreover, some Mg_17_Al_12_ phase in the form of plates can be observed within the SiC nanoparticle dense zones as shown in Figure 8 and Figure 9.

**Figure 10:**
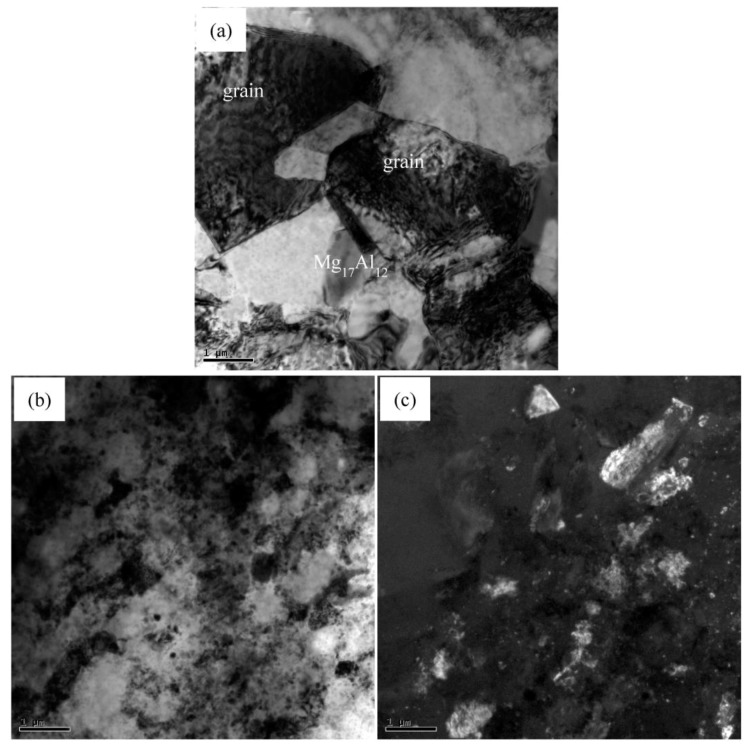
TEM micrographs of nano-SiCp/AZ91 composite after 6 passes at 400 °C and 3 passes at 350 °C: (**a**) DRX grains and precipitated phase; (**b**) bright field image and (**c**) dark field image of distribution of SiC nanoparticles.

Figure 10 gives the TEM images of nano-SiCp/AZ91 composite after 6 passes at 400 °C and 3 passes at 350 °C. As can be seen from Figure 10a, the recrystallized grains as well as the second phase Mg_17_Al_12_ exist in the nanoparticle sparse regions after 6 passes at 400 °C and 3 passes at 350 °C. Besides, due to the accumulative strain introduced by multi-pass forging, dislocation density is high inside some DRX grains. In nanoparticles dense zones, the distribution of SiC nanoparticles is relatively uniform and the dislocation density is low according to TEM bright field images (Figure 10b). TEM dark field image indicates that the recrystallization grain size within the SiC nanoparticles dense zones was significantly reduced compared with that in the nanoparticle sparse regions, as given in Figure 10c. Moreover, the SiC nanoparticles are distributed in the vicinity of the DRX grains, indicating that the addition of SiC nanoparticles can refine grain.

**Figure 11:**
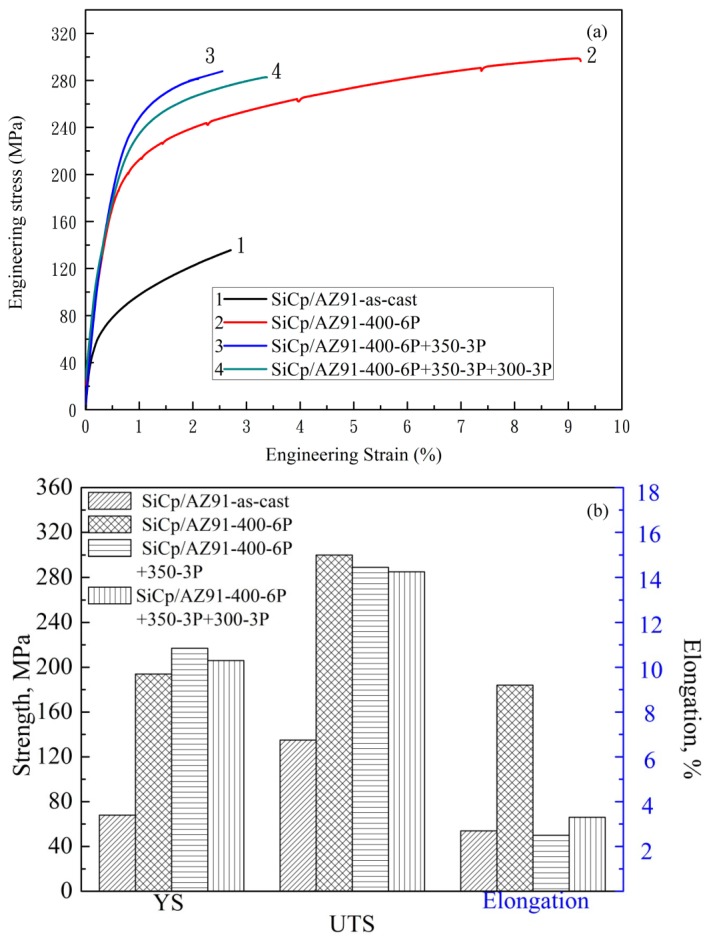
(**a**) The engineering stress–engineering strain curves and (**b**) tensile strength of the nano-SiCp/AZ91 composites after multi-pass forging with varying temperatures.

Figure 11 gives the engineering stress-engineering strain curves and tensile strength of the nano-SiCp/AZ91 composites after multi-pass forging with varying temperatures. In comparison to those of its as-cast counterpart, the composite strength is remarkably increased after multi-pass forging with varying temperatures. The tensile yield strength of the nano-SiCp/AZ91 composites after 6 passes at 400 °C, 3 passes at 350 °C is increased as compared to that after 6 passes at a constant temperature of 400 °C. It is considered that the main reinforcing mechanism of metal matrix composites are grain refinement strengthening, Orowan strengthening, dislocations strengthening and load bearing [37]. Based on our previous study [38], the strengthening mechanisms are mainly grain refinement strengthening and Orowan strengthening for the as-cast nano-SiCp/AZ91 composites. In the present work, as shown in Figure 6, with the increase of forging passes and the decrease of deformation temperature matrix structures are gradually refined, which can contribute to the yield strength (YS) enhancement of the composites. The precipitated second phases with large amounts and SiC nanoparticles are harder than matrix alloy under ambient condition. During the tensile test, dislocations of the composite need to across the grains, and can be inhibited by the nanoparticles and precipitate phases [39]. The tensile properties of the composite can be increased by this Orowan strengthening effect. However, the ultimate tensile strength (UTS) and elongation to fracture after multi-pass forging with varying temperatures are smaller than that after 6 passes at constant temperature of 400°C. This is because coarse second phase with small amount are distributed along grain boundaries in the nanoparticle dense zone (Figure 7b,c), and easy to be broken during a uniaxial tensile test at room temperature. Moreover, although the grains in the composite are further refined with the increase of the forging passes, the YS of the composite after 6 passes at 400 °C, 3 passes at 350 °C and 3 passes at 300 °C exhibits slight decrease compared with that after 6 passes at 400 °C, 3 passes at 350 °C. This could be attributed to texture softening caused by the rotation of (0002) basal planes, which may impair mechanical properties [26,35]. In addition, when the temperature range of the multi-pass forging is the same, the yield strength of the composites after 6 passes at 400 °C, 3 passes at 350 °C is improved compared with that after 3 passes at 400 °C, 3 passes at 350 °C and 3 passes at 300 °C [36]. This can be attributed to the increase of initial forging passes under constant temperature is helpful for improving the distribution of SiC nanoparticles, which can promote grain refinement and Orowan strengthening.

## 4. Conclusions

The microstructures and tensile properties of nano-SiCp/AZ91 composites containing SiC nanoparticle and second phases Mg_17_Al_12_ have been investigated in the paper, and the main conclusions can be described as follows:

(1) The distribution of SiC nanoparticles is improved while the amount of dispersed nanoparticles is increased with the increase of single-pass forging temperature for the present composite, resulting in an increased DRX degree and refined grains.
(2) SiC nanoparticle dense zones with twins may deform by twinning in the matrix while nanoparticle sparse zones with deformation bands and high density dislocations deform mainly by dislocation slip.
(3) Yield strength and ultimate tensile strength are gradually enhanced with the increase of the single-pass forging temperature from 300 °C to 400 °C. This indicates that an initial high forging temperature is beneficial to improve the tensile properties.
(4) During multi-pass forging with varying temperatures, the value of grain size is gradually reduced and the distribution is more even with increasing the forging passes and lowering the deformation temperature. The amount of the precipitated second phases is significantly increased compared with that after multi-pass forging at a constant temperature.
(5) The improvement in yield strength could be attributed to grain refinement strengthening and Orowan strengthening, which are related to synergistical enhancement by precipitated second phases and SiC nanoparticles.
